# Angiotensin receptor blockade with Losartan attenuates pressor response to handgrip contraction and enhances natriuresis in salt loaded hypertensive subjects: a quasi-experimental study among Nigerian adults

**DOI:** 10.11604/pamj.2019.34.188.18317

**Published:** 2019-12-09

**Authors:** Francis Muyiwa Agbaraolorunpo, Olusoga Adekunle Sofola, Chikodi Nnanyelu Anigbogu, Elaine Chinyelu Azinge

**Affiliations:** 1 Department of Physiology, College of Medicine, University of Lagos, Lagos, Nigeria; 2 Department of Clinical Pathology, College of Medicine, University of Lagos, Lagos, Nigeria

**Keywords:** Handgrip contraction, sodium excretion, vascular resistance, losartan, hypertensive Nigerians, maximum voluntary contraction

## Abstract

**Introduction:**

Sympathetic and Renin-Angiotensin-Aldosterone systems play crucial roles in blood pressure response to increased salt intake. This study investigated the effects of angiotensin receptor blocker (ARB) and sympathetic excitation on the responses of blood pressure (BP) and peripheral vascular resistance (PVR) in salt loaded normotensive (NT) and hypertensive (HT) Nigerian subjects.

**Methods:**

16 NT and 14 HT participants, that were age-matched [39.9 ± 1.3 vs 44.1±2.1yrs (P= 0.10)], underwent 5 days each of oral administration of 200mmol NaCl, and 200mmol NaCl + 50mg Losartan, preceded by a baseline control condition. BP and PVR responses to 30% Maximum Voluntary Contraction (MVC) of handgrip (HG) for one minute were determined at baseline, after salt load and after salt + Losartan. Data were presented as Mean ± SEM, and analyzed with two-way ANOVA and paired t-test, with P<0.05 accepted as significant.

**Results:**

BP and PVR were significantly increased by HG at baseline, after salt load and after salt + Losartan in NT and HT. Salt load augmented the HG-induced SBP (P=0.04) and MABP responses (P=0.02) in HT. While Losartan attenuated the HG- induced Systolic Blood Pressure (SBP) SBP response (P=0.007) and DBP response (P=0.003) in HT and NT respectively after salt + Losartan. HG-induced PVR response was significantly accentuated after salt load in HT (P=0.005), but it was not significant in NT (P=0.38).

**Conclusion:**

The implication of our finding is that angiotensin II receptor blockade possibly attenuates salt-induced sympathetic nerve excitation in black hypertensive patients.

## Introduction

Studies have shown that salt intake plays a key role in the regulation of blood pressure [1] and in the pathophysiology of hypertension [2,3] Sympathetic Nervous System (SNS) and Renin Angiotensin Aldosterone System (RAAS) are both involved in short and long term regulation of blood pressure respectively [4,5]. Abnormal regulation of these dual systems by salt may result in hypertension. Unfortunately, industrialization has led to an astronomical rise in the amount of dietary salt consumption, with the value increasing markedly from less than 0.25g/day consumed several years ago, to a more disturbing level of 9-12g/day [6]. Meanwhile, genetic variations have been reported to exist in the responses of blood pressure of an individual to high salt diet [7]. In particular, blood pressure of salt sensitive individuals increased significantly following high salt diet, but decreased after low salt diets, while the blood pressure of salt resistant individuals respond less significantly to a high salt diet [8]. In addition, several studies have also demonstrated that there is an existing nexus between salt sensitivity and hypertension [9,10]. However, just as dietary salt consumption is increasing globally, the prevalence of hypertension is also soaring in Nigeria and in other black communities. For instance, the prevalence of hypertension rose from 16%, as reported by Cooper in 1997 [11], to values around 32.8% and 36.6%, between the year 2008 and 2012 [12,13].

Similarly, salt sensitivity has been reported to be high among normotensive and hypertensive Nigerians, with the prevalence put at 52% and 61% respectively [14].The involvement of renal Epithelial Sodium Channels (ENaC) in the pathophysiology of salt sensitive hypertension has also been suggested and demonstrated among Nigerians [15]. Other experimental studies have also attempted to elucidate the mechanism underlying salt-induced hypertension [16]. Interestingly, the modulation of SNS and RAAS activities by salt have been suggested as plausible mechanisms underlying the development of hypertension [17]. Presently, the role of angiotensin receptor inhibition in salt mediated changes in blood pressure is controversial, in particular, among the black population. This is of special interest as angiotensin II receptor blockers have been reported to be of lesser benefit in the control of hypertension among the black population [18]. However, Mabayoje and Oke (2004) [19] in their clinical trial of monotherapy with Telmisaltan, an angiotensin II receptor blocker, in hypertensive patients, reported the efficacy of the drug in the control of hypertension among the black population. Handgrip isometric exercise test is an autonomic function test similar to cold pressor test used in the evaluation of sympathetic activity. This test has been used in unmasking the tendency of developing hypertension in high risk individuals [20]. Handgrip contraction results in the stimulation of the cardiovascular system through the sympathetic nervous pathway [21]. Recently, the effects of sympathetic nervous activation using cold pressor test did indicate blunted responses in hypertensive Nigerians [22]. It is therefore important to further investigate the role of the interaction between sympathetic and RAAS in the development of hypertension with regards to renal handling of salt among Nigerians.

## Methods

The procedure for the study was approved by the Ethics Committee of College of Medicine of the University of Lagos (CM/COM/08/VOL.25). Informed written consent was obtained from the volunteers recruited into the study. The study conformed to the standards outlined in the Declaration of Helsinki (2008) [23].

**Subjects' selection:** forty-four male volunteers aged between 25 and 65 years, from Lagos South West Nigeria, were screened for the study. Eligible age-matched 16 normotensive (NT, M=14, F=2) and 14 hypertensive subjects (HT, M=13, F=1) were then enlisted to participate in the study. Participants were the volunteer students and workers of College of Medicine of the University of Lagos and the adjoining community. The study was conducted at the Cardiovascular Research Laboratory of the Department of Physiology of College of Medicine of the University of Lagos. Data were obtained between 8am and 12 noon on the day of the experiment. Normotension was defined as Systolic Blood Pressure (SBP) and Diastolic Blood Pressure (DBP) less than 140mmHg and 90mmHg respectively, while hypertension was defined as SBP and DBP consistently equal to or greater than 140mmHg and 90mmHg on two occasions. Inclusion criteria for cases of hypertension were SBP≥140mmHg but < 160mmHg and DBP≥ 90mmHg but < 100mmHg in newly diagnosed hypertensive subjects, while exclusion criteria include any history of end organ diseases and chronic metabolic conditions and musculoskeletal disorders, pregnancy and lactation. Subjects were asked to refrain from alcohol, caffeine and exercise 24hrs before the study. 24-hour urine collection was also carried out.

**Study design:** the study was a quasi-experiment with groups' comparison design. The subjects were assigned into two groups namely normotensive and hypertensive on the basis of their SBP and DBP. The subjects were group matched for age, sex and BMI in order to remove potential confounders between the two groups. The effect of salt load was evaluated before and after losartan ingestion in each group, and compared between the two groups. The subjects' baseline Mean Arterial Blood Pressure (MABP), Heart Rate (HR), Peripheral Vascular Resistance (PVR), plasma Sodium ion (Na+) and Potassium ion (K+) concentration, 24hr Urinary Sodium ion (Na+) and Potassium ion (K+) concentrations were determined initially at the beginning of the study. The subjects were then instructed to take salt (NaCl) containing 200mmol of Na+ for 5 days [14], and their corresponding MABP (mmHg), PVR (mmHg/m/s), plasma Na+ and K+, urinary Na+ and K+ were repeated on the morning of the 6th day. This was followed by a one-week period of salt wash out, when the subjects were taken off salt. Next, the subjects were instructed to ingest the same quantity of salt as well as 50mg losartan daily for 5days, with the Losartan (procured from Ranbaxy Nigeria Ltd.) repeated on the morning of the experimental day. MABP and PVR were further evaluated on the morning of the 6th day of the experiment following salt and Losartan administration. In addition, responses of MABP, HR and PVR to one-minute handgrip at 30% maximum voluntary contraction (MVC) in kilogram (kg) were determined prior to salt ingestion, after salt ingestion, and after a combination of salt ingestion and Losartan. The salt was packaged in drug sachets, and was taken in two divided doses with 500ml of water in the morning and at night. To ensure compliance, 24-hour urinary output and urinary electrolytes were used to assess salt intake compliance [24].

**Blood pressure and PVR measurement:** Blood Pressure (BP) was determined with a mercury Sphygmomanometer (Accoson model) taking the mean of two close consecutive readings, following a 15- minute period of rest in a sitting position. Finger blood flow was determined with finger plethysmography by AD instrument Power Lab [25], with the subject in sitting position following a 15 minutes period of rest, and after one minute of handgrip at 30% MVC. MABP was calculated from DBP + 1/3PP (Pulse Pressure), while PVR was calculated as MABP/FBF (mmHg/ml/s). The readings were made prior to salt loading, after salt loading and after salt + losartan administration.

**Finger Plethysmography:** finger blood flow wasdetermined by a venous occlusion plethysmography, using a finger plethysmography [26]. A flow pattern similar to forearm venous occlusion was obtained with an initial slope, followed by a plateau, and a falling slope. The initial slope represents the rate of blood flow in ml/sec. Calibration was done by setting the AD instrument PowerLab to 100/s sample rate; y-axis at 0.1V to 1ml and 1V to 10 ml.

**Heart rate:** heart rate was determined from lead II using a bipolar 3-lead ECG and from the pulse oximeter of the AD instrument PowerLab.

**Handgrip test**: MVC was determined with a manual hand dynamometer (Camry model). The participants squeezed the dynamometer with their dominant hand, with maximum effort sustained for three to four seconds. This was determined twice and the average taken as the MVC. Thereafter, the subjects were instructed to compress the handle of the dynamometer at 30% of MVC for one minute with the sphygmomanometer cuff positioned on the contralateral arm. SBP, DBP, HR and FBF were determined at the end of one minute, with the handgrip still sustained.

**24-hr urine and blood collection:** 24-hr urine excretion was collected according to the method described by Elias *et al*. (2011) [15]. 5ml of aliquot of urine was then taken and stored in a refrigerator at -20^o^C until it was analyzed for K+ and Na+ concentrations. Urinary sodium excretion was evaluated before and after salt-loading [27] and after salt + losartan. Urinary chemistry analysis for K+ and Na+ was carried out with automated ion selective Electrode analyzer (ISE Pack SFRI Sari,Lie dit Bergaton). The 24 hr sodium excretion value (mmol/24hrs) was calculated as the concentration of sodium in the urine (mmol/L) multiplied by the 24hr-urinary volume (L). Blood was collected by venipuncture into heparinized bottles, centrifuged at 3000rpm for 10 minutes and stored at -80^o^C in deep freezer before analysis. Analysis was carried out with ISE 6000 using the auto analyzer.

**Data Analysis:** data are presented as Mean ± SEM. MABP and PVR responses to handgrip were expressed as percentages. The statistical difference between the rest and handgrip data were analyzed using Paired-t test, while the statistical comparisons of the timelines variables in each group and between the groups were performed using two-way analysis of variance (ANOVA), followed by Tukey post hoc. Statistical significance was accepted at P<0.05. GraphPad Prism 7 package was used for the analysis.

## Results

**Biodata:** as shown in (Table 1), the mean age of the normotensive (NT) (n=16; M=14, F=2) and the hypertensive (HT) (n=14; M=13, F=1) subjects were 39.9 ± 1.3 years and 44.1 ± 2.1 years respectively (P=0.10). The mean heights (P=0.32), weights (P=0.29) and BMI (P=0.81) of both groups were not significantly different.

**Table 1:** Showing age, height and weight of subjects who participated in the study

subjects	Age (years)	Weight (kg)	Height (cm)	BMI (kg/m^2^)
Normotensive (n=16)	39.9 ± 1.3	71.69 ± 2.28	168.3 ± 2.72	25.62 ± 1.20
Hypertensive (n=14)	44.1 ± 2.1	70.91 ± 2.15	170.7 ± 2.00	24.35 ± 0.69
P value	0.10	0.29	0.32	0.81

Data are presented as Mean ± SEM, with***P<0.0001 vs MABP_Rest_ are significant for NT and HT

**Figure 1:**
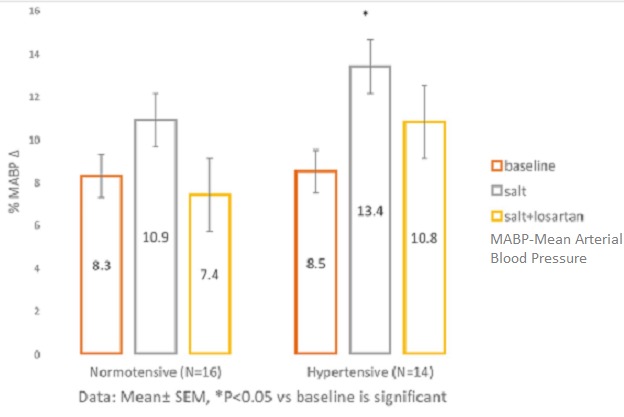
MABP response to handgrip contraction

**MABP responses to Handgrip contraction after salt-loading and after salt + losartan administration in NT and HT** (Table 2)**:** shows that in both groups, MABP increased significantly (P<0.001) in response to HG. In the NT, MABP increased from baseline by (8.25 ± 1.58)mmHg in response to HG; by (10.88 ± 2.21)mmHg after salt load, and by (7.39 ± 2.25)mmHg after salt + losartan. Similarly, in the HT, MABP increased significantly by (8.50 ± 1.48)mmHg from baseline, by (13.43 ± 1.29)mmHg after salt ingestion, and by (9.79 ± 1.77)mmHg, after salt + losartan. (Figure 1) shows that the HG-induced MABP increase was significantly augmented (P=0.02) after salt load in the HT, but was maintained (P=0.29) in the NT. After salt + losartan, there was a non-significant trend towards a lower MABP in the NT group (0.072), while the response in HT group was not statistically significant (P=0.23).

**Table 2:** Showing the response of MABP to handgrip contraction after salt loading and after salt + losartan in normotensive and hypertensive subjects

Groups	Normotensive (n=16)			Hypertensive (n=14)		
MABP (mmHg)	baseline	salt	Sal+losartan	baseline	salt	Salt+losartan
Rest	89.56 ± 1.49	93.19 ± 1.67	88.77 ± 1.26	111.1 ± 1.24	110.9 ± 2.36	109.9 ± 1.69
Handgrip	97.81 ± 2.07***	104.7 ± 2.66***	96.15 ± 2.73***	120.9 ± 2.07***	124.4 ± 2.31***	120.6 ± 2.08***
MABP Δ	8.25 ± 1.58	10.88 ± 2.21	7.39 ± 2.25	8.50 ± 1.48	13.43 ± 1.29	9.79 ± 1.77

Data are presented as Mean ± SEM,

*** P<0.0001 is significantly higher than MABP at Rest in Normotensive and Hypertensive subjects

**Figure 2:**
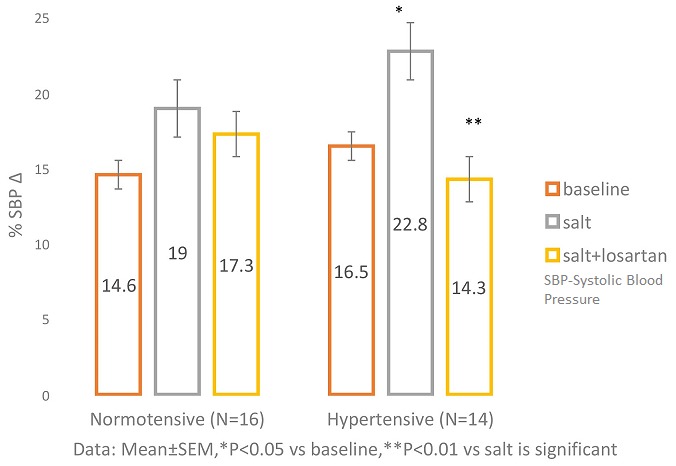
SBP response to handgrip contraction

**SBP responses to Handgrip contraction after salt loading and after salt + losartan administration in NT and HT** (Figure 2)**:** shows that in NT, SBP increased by (14.56 ± 2.0)mmHg in response to HG at baseline; by (19.00 ± 2.23)mmHg after salt load, and by (17.13 ± 2.06)mmHg after salt + losartan. The corresponding values in response to HG in HT was (16.50 ± 2.72)mmHg at baseline, (22.79 ± 3.15)mmHg after salt load, and (14.07 ± 2.41)mmHg after salt + losartan. The HG-induced increase in SBP was significantly accentuated (P=0.04) by salt load, and was also significantly attenuated (P=0.007) after salt+ losartan in the HT. However, the response was not significantly affected by salt load (P= 0.21) and salt + losartan (P=0.75) in the NT group.

**Figure 3:**
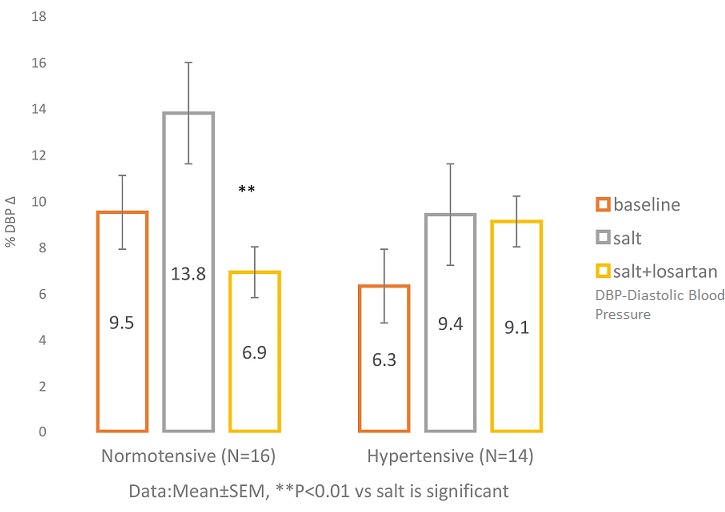
DBP response to handgrip contraction

**DBP responses to Handgrip contraction after salt loading and after salt + losartan administration in NT and HT** (Figure 3)**:** shows that in response to HG in NT, DBP increased by (9.50 ± 1.27)mmHg at baseline, by (13.75 ± 2.37)mmHg after salt load, and by 6.94 ± 1.38 after salt + losartan. Similarly, in HT, DBP increased by (6.29 ± 2.01)mmHg at baseline, by (9.39 ± 1.56)mmHg after salt load, and by 9.08 ± 1.89 salt + losartan. DBP response to HG after salt load showed a trend towards higher DBP (P=0.075) in the NT group but not (P=0.40) in the HT. Furthermore, the HG-induced DBP increase was reduced significantly (P=0.003) after salt + losartan in NT, but was however maintained (P=0.99) in HT.

**Figure 4:**
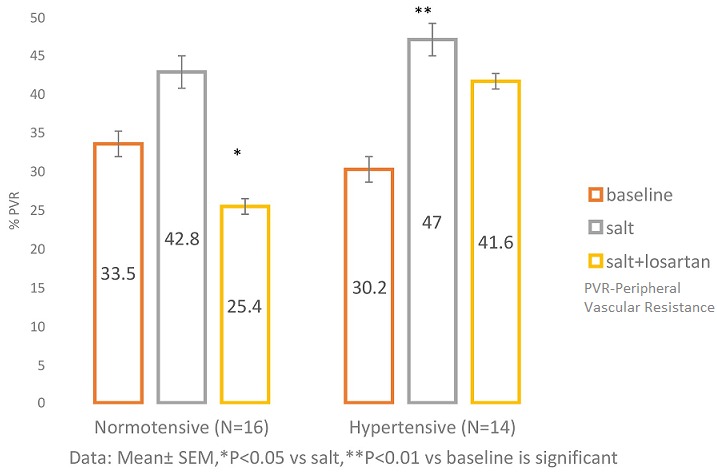
PVR response to handgrip contraction

**PVR responses to Handgrip contraction after salt-loading and after salt + losartan administration in NT and HT:** (Figure 4) shows that in NT, PVR was increased significantly by 33.53 ± 3.70% (P=0.0005) at baseline, by 42.84 ± 5.04% (P= 0.0007) after salt load, and by 25.40 ± 2.67 % (P=0.003) after salt + losartan. Similarly, in HT, PVR increased significantly in response to HG by 30.19 ± 5.39% (P=0.005) at baseline, by 47.02 ± 4.26% (P=0.03) after salt load, and by 41.57 ± 5.33% (P=0.005) after salt + losartan, The HG-induced increase in PVR was significantly augmented (P=0.005) by salt load in the HT, while it was slightly but not significantly enhanced (P=0.38) in the NT. However, the response was significantly attenuated (P=0.02) after salt +losartan in the NT, while it was slightly reduced (P=0.25) after salt + losartan.

**HR responses to Handgrip contraction after salt loading and after salt + losartan administration in NT and HT:** in NT, there was no significant change in HR in response to HG at baseline (69.50 ± 2.15 vs 71.75 ± 2.27), after salt load (70.20 ± 1.99 vs 71.93 ± 1.96) and after salt + losartan (70.43 ± 2.86 vs 71.57 ± 2.70). Similarly, in HT, HR response to HG remained unchanged at baseline (70.57 ± 2.24 vs 71.00 ± 2.25), after salt-loading (69.29 ± 2.38 vs 70.36 ± 2.36) and after salt + losartan administration (69.86 ± 1.89 vs 72.00 ± 2.16).

**Response of Plasma Na+ and K+ concentration to salt loading and losartan administration in NT and HT** (Table 3)**:** shows that Plasma Na+ increased significantly following salt loading in both groups. In NT, it increased from 136.9 ± 0.42 to 139.8 ± 0.39mmol/L (P = 0.01) after salt loading, while in HT, it increased from 135.6 ± 0.75 to 141.0 ± 0.99mmol/L (P = 0.0001). This was followed by a significant decrease to (136.6 ± 1.14)mmol/L (P = 0.0001) after salt + Losartan in the NT, and to (135.8 ± 0.94)mmol/L (P<0.0001) in the HT. However, plasma K+ remained relatively unchanged in the two groups after salt ingestion and after salt + losartan administration.

**Table 3:** Showing the effect of salt loading and losartan on plasma and urinary sodium, potassium and urinary volume

Parameter	Normotensive (n=16)			Hypertensive (n=14)		
	baseline	salt	salt+losartan	baseline	salt	salt+losartan
plasma Na^+^	136.9* ± 0.42	139.8 ± 0.39	136.6 ± 1.14***	135.7 ± 1.08^###^	141.0±1.00	135.8±0.94^####^
plasma K^+^	4.07 ±0.12	4.02 ± 0.11	4.10 ± 0.12	3.96±0.10	3.94±0.07	3.75±0.08
UNa+V(mmol/24hrs	165.5 ±8.0	255.0±23.5***	255.8±20.1***	118.8±17.76	234.3 ± 26.45 ^####^	267.0 ± 27.43 ^####^
U_K+_V(mmol/24hrs)	20.56±2.9	22.9±3.6	25.6±3.2	18.9 ± 2.2	25.2 ± 3.7	21.75 ± 1.93
24hrs-Urinary Volume (V)(ml)	1521 ± 114.7	1838 ±128.3**	1802 ± 89.9	1404 ± 143.4	1722 ± 184.8	1685 ± 165.8

Plasma Na+:

* P < 0.05 vs salt

*** P < 0.001 vs salt (NT);

### P<0.001

#### P<0.0001 vs salt (HT) are significant

U_Na_^+^ V(mmol/24hrs):

***P<0.001 vs baseline(NT)

Urinary volume**P<0.01(NT)

^####^P<0.0001 vs baseline (HT)

**Response of urinary Na+ and K+ excretion to salt loading and losartan administration in NT and HT** (Table 3)**:** shows that urinary sodium excretion increased significantly in NT (P= 0.0002) and in HT (P<0.0001) after salt ingestion. Specifically, urinary sodium excretion (UNa+V) increased from (165.5 ± 8.0)mmol/24hrs at baseline to (255.0 ± 23.5)mmol/24hrs. After salt load in NT; and from 118.8 ± 17.76 at baseline to (234.3 ± 26.45)mmol/24hrs, after salt load in HT. In comparison with salt load, this was followed by a non-significant (P=0.98) increase to (267.0 ± 27.43)mmol/24hr after salt + losartan in HT, and was maintained (P=0.32) at (255.8 ± 20.1)mmol/24hrs after salt + losartan in NT.

## Discussion

In this present study, MABP and PVR increased significantly in response to one-minute hand grip, prior to salt loading, after salt loading, and following a combination of salt load and losartan in both the NT and the HT. Generally, handgrip contraction facilitates sympathetic outflow [28] to bring about these pressor responses, but these responses were modulated by salt load and losartan in this current study. Specifically, MABP and SBP responses to handgrip (HG) were significantly augmented by salt loading in the hypertensive group but less significantly in the normotensive group. This is in agreement with the findings of Ishii *et al*. (1983) [29] which demonstrated that pressor responses to isometric exercise were significantly greater in the HT group when compared with the NT group following salt loading. An earlier study had also indicated that hypertensive adults’ exhibit exaggerated sympathetic and pressor responses to handgrip exercise [30]. Meanwhile, pressor responses to handgrip decreased significantly following a combination of salt + losartan administration, with a significant attenuation of SBP and DBP observed in the HT and the NT subjects respectively. Noticeably, this resulted in moderate attenuation of MABP in the NT and the HT. A similar study in patients with diabetic nephropathy suggests that angiotensin II receptor blockade reduces salt sensitivity of blood pressure [31]. However, our result is contradictory to a previous study in healthy men, that suggested that short-term ARB (1 week 100mg Losartan) did not attenuate reflex sympathoneural responses to HG of 30% MVC for 2 min [32].

This may be due to racial differences and handgrip duration. Meanwhile, the inhibition of angiotensin type I receptor by ARB during salt loading partly mitigated sympathetic nervous activities, and this could have contributed partly to the observable reduction in the SBP, DBP and MABP responses to HG in this study. In support of this, a study suggested that ARBs block central and peripheral sympathetic nerve activity in a rat model of neurogenic hypertension [33]. Furthermore, we observed that salt ingestion significantly enhanced PVR responses to handgrip in the HT group, while losartan significantly decreased the handgrip-induced increased PVR response in salt loaded NT subjects with no significant effect in the HT. This is not in line with a previous study that showed that administration of eprosartan, an ARB, resulted in a decrease in PVR in hypertensive rats fed on high-salt and high-fat diets [34]. Also, in this present study, we observed that in response to salt load, plasma sodium increased significantly in the NT and the HT. This is in conformity with earlier reports that sudden increase in dietary sodium results in noticeable increase in plasma sodium concentration [35,36]. The reason for this higher plasma sodium in hypertensive patients has been attributed to possible sodium retention secondary to impaired ability of the kidney to excrete sodium ions [37]. Consequently, the elevated plasma sodium will result in fluid retention and extracellular fluid volume expansion, that can compound or contributes to the hypertension. Importantly, the elevated plasma sodium observed in this study was significantly reduced by losartan following the concurrent ingestion of salt and losartan, in both the hypertensive and normotensive subjects. This is in keeping with the natriuretic effect of angiotensin II receptor blockers (ARB) that have been reported in experimental animal models [38]. Meanwhile, plasma K+ remained unchanged after salt load and after a combination of salt and losartan in this study. Our results also show that, mean urinary sodium excretion increased significantly after salt ingestion in both the normotensive and the hypertensive subjects, with significantly higher sodium excretion observed in the normotensive group. The explanation for this observation may be that hypertensive individuals exhibited higher tendency to retain dietary sodium when compared with normotensive individuals.

Following a combination of salt and losartan ingestion, urinary sodium excretion increased markedly but not significantly among the hypertensive subjects. Also in an earlier study, Burnier *et al*. (1993) [39] reported natriuretic effect of ARB in both normotensive and hypertensive subjects during salt depletion. In this current study, urinary K+ excretion remained unchanged following salt load and losartan. The mechanisms adduced for the renal antinatriuretic action of Angiotensin II include its effect on renal tubular sodium transporters [40]. This antinatruiretic action is apparently inhibited by Losartan, resulting in natriuresis, contraction of ECF volume and reduction in blood pressure. These natriuretic actions of Losartan may include the inhibition of ENaC expression [41] and activity [42], as well as the suppression of renal sympathetic activity [43]. Therefore, the lowering of plasma sodium concentration and the enhancement of sodium excretion by ARB may both reduce salt-induced increased cardiac output and blood pressure responses to handgrip activity independent of PVR modulation. This view supports an earlier study which reported a marked increase in cardiac output, with no change in systemic vascular resistance in response to salt load in salt sensitive patients [44]. Therefore, our current findings provide evidence in support of the plausible mechanism underlying the action of AT1 receptor inhibition in blood pressure regulation among black hypertensive patients. However, the weaknesses of this study include lack of direct evaluation of sympathetic activity during the handgrip test and the lesser participation of female subjects in the study.

## Conclusion

Salt ingestion significantly augmented pressor response to handgrip contraction in HT comparably with NT, and this was ameliorated by losartan in NT via PVR modulation, but independent of PVR modulation in HT. However, Losartan enhanced natriuresis in the HT. Our findings suggest that high salt diet possibly accentuates sympathetic nervous activities in black hypertensive patients and salt sensitive normotensive black. While, angiotensin II receptor blockade likely mitigates salt-induced sympathetic nerve excitation in black hypertensive patients, with possible blood pressure control independent of PVR modulation. Further studies will be required in this regard among the black, to establish the potential inhibitory action of AT1 receptor blockers on sympathetic nervous system in salt sensitive individuals, by directly evaluating sympathetic nerve activities.

### What is known about this topic

- Handgrip contraction activates muscle sympathetic nervous activity;
- Pressor response to handgrip is exaggerated in salt loaded hypertensive patients.

### What this study adds

- Salt loading significantly augmented hangrip pressor response via peripheral vascular resistance modulation in hypertensive subjects;
- Losartan significantly attenuated handgrip-induced pressor response in salt loaded hypertensive, independent of peripheral vascular resistance modulation.

## Competing interests

The authors declare no competing interests.
